# Effects of TiO_2_ Nanoparticle Addition on Microstructure and Selected Properties of Ag–xTiO_2_ Composites

**DOI:** 10.3390/ma14174846

**Published:** 2021-08-26

**Authors:** Anna Wąsik, Beata Leszczyńska-Madej, Marcin Madej

**Affiliations:** 1 Department of Materials Science and Non Ferrous Metals Engineering, Faculty of Non Ferrous Metals, AGH University of Science and Technology, 30 Mickiewicza Ave., 30059 Cracow, Poland; anna.wasik@agh.edu.pl; 2 Department of Physical and Powder Metallurgy, Faculty of Metals Engineering and Industrial Computer Science, AGH University of Science and Technology, 30 Mickiewicza Ave., 30059 Cracow, Poland; mmadej@agh.edu.pl

**Keywords:** Ag–xTiO_2_ composites, powder metallurgy, microstructure investigations, silver, TiO_2_ nanoparticles

## Abstract

The paper presents the results of a study of the microstructure and selected properties of silver-based composites reinforced with TiO_2_ nanoparticles, produced by the powder metallurgy method. Pure silver powders were mixed with TiO_2_ reinforcement (5 and 10 wt%) and 5 mm steel balls (100Cr6) for 270 min in a Turbula T2F mixer to produce a homogeneous mixture. The composites were made in a rigid die with a single-action compaction press under a pressure of 400 MPa and 500 MPa and then sintered under nitrogen atmosphere at 900 °C. Additionally, to improve the density and mechanical properties of the obtained sinters, double pressing and double sintering operations were conducted. As a result, compacts with a density of 90–94% were obtained. The microstructure of the sintered compacts consists of uniform grains, and the TiO_2_ reinforcement phase particles are located on the grain boundaries. There were no discontinuities at the Ag–TiO_2_ contact boundary, which was confirmed by SEM and TEM analysis. The use of a higher pressure had a positive effect on the hardness and flexural strength of the tested materials. It was found that the composites with 5 wt% TiO_2_ pressed under 500 MPa are characterized by the highest level of mechanical properties. The hardness of these composites is 57 HB, while the flexural strength is 163 MPa.

## 1. Introduction

Metal–ceramic composites have attracted much attention due to their extraordinary properties such as high strength, low weight, high fatigue strength or electrical properties when compared to traditional materials [1,2]. However, the final properties of the composite material are greatly affected by the method of manufacturing, component composition and weight/volume fraction of individual components and, in particular, depend on the amount and size of the introduced reinforcement particles since it is the size of the particles that determines the reinforcement mechanism [3]. Pure silver is an excellent conductor of heat and electricity [4]; nonetheless, introducing reinforcing particles affects its thermal conductivity. Wieczorek et al. [5] showed that the presence of a reinforcing phase in the form of Al_2_O_3_ and SiC particles leads to a decrease in the thermal conductivity in relation to pure silver. Silver is also characterized by high plasticity, ductility and chemical durability [6]. To improve its strength properties and wear resistance, reinforcing phase particles are introduced into the silver matrix [7]. For example, Rigou et al. [8], in their study, added diamond particles as suspension to the plating silver bath to improve the sliding wear behavior of silver-plated surfaces, which are normally characterized by low hardness and poor wear resistance. The results show that the surface of the silver coating containing diamond particles exhibited low values of the coefficient of friction and wear rate.

Silver matrix composites can be classified according to the type of dispersed phase, which may be a pure element or carbide and oxides. Very common reinforcement particles for silver matrix composites are oxides such as CdO, SnO_2_, ZnO, Al_2_O_3_ and TiO_2_ [9]. Among them, TiO_2_ has attracted great attention as a reinforcing phase due to its high chemical stability and low cost [10]. The application of TiO_2_ particles as a reinforcement increases the hardness, wear and corrosion resistance of the base material [11,12]. However, to fulfill its function properly, the reinforcement phase has to be homogeneously distributed in the matrix material. Haoa et al. [13] fabricated a silver matrix composite reinforced with Ag-doped graphene nanosheets using powder metallurgy methods. Their results indicate that the silver matrix composite, due to the homogeneous dispersion of Ag-doped graphene, has good comprehensive properties at 0.5 wt% Ag-doped graphene, in which electrical conductivity reaches up to 98.62% IACS. Thanks to the mentioned properties, silver matrix composites have found application in the electronic packing industry as contact materials in electronics and electrical brushes [14,15,16].

Silver, additionally, has antimicrobial and photocatalytic activity; therefore, it has found its use in biomedical applications [17]. The antibacterial properties of composites with a silver matrix have also been tested by many authors. Yen-Wen Wanga et al. [18] obtained macroporous Ag–TiO_2_ composite foams which exhibited strong antibacterial activity against *Escherichia coli* bacteria. The antibacterial activity of silver matrix composites makes them a material suitable for antibacterial and photocatalytic activities that can be successfully used in chemical filtration applications. Nanocomposite systems with high photoactivity, such as silver-modified TiO_2_, are employed as electrodes for dye degradation [19,20]. Youngmi Koo et al. [21], in their research, indicate that Ag–TiO_2_–CNT composite nanoparticles can be used in environmental protection as a factor enabling the breakdown of organic pollutants.

Robert Liang et al. [22] investigated the alternative application of Ag–TiO_2_ thin film composites in the photocathodic protection of stainless steel, while improving the degradation of pollutants under photocatalysis. The authors noted that corrosion in moist chloride-containing environments is still a concern as the passive film formed on the surface of stainless steel is broken down. A potential solution to this problem is photocathodic protection. TiO_2_ is commonly used as a photoanode material [23]. However, the authors indicated that it requires ultraviolet light and rapidly undergoes charge re-combination. Therefore, metal nanoparticles, such as silver, might be added. In the above-mentioned work [22], the authors deposited Ag nanoparticles in a silica–titanium coating with the addition of TiO_2_ onto glass by electrophoretic deposition. This solution allowed minimization of the weight loss as a result of corrosion processes and surface oxidation.

As was mentioned above, manufacturing methods have a significant effect on the final properties of composite materials as they have to provide uniform distribution of the reinforcement in the matrix material. Various methods can be used to produce metal matrix composites and may be based on casting methods or the powder metallurgy technique [24]. This study thus investigated the effect of a TiO_2_ nanoparticle addition on the densification, microstructure and selected properties of Ag–xTiO_2_ composites prepared by conventional powder metallurgy techniques. Particular attention was paid to the interface of the Ag matrix and the reinforcement phase as well as to the phenomena occurring in the material during the manufacturing process, including the stability of the TiO_2_ phase.

## 2. Materials and Methods

**Figure 1:**
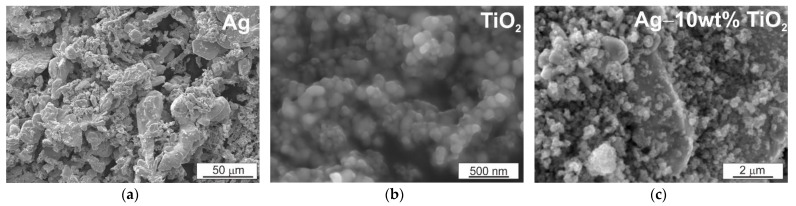
Powder morphology: (**a**) Ag (0–63 µm); (**b**) nano TiO_2_; (**c**) Ag–10 wt% TiO_2_; SEM.

The research material was electrolytic silver powders with a particle size below 63 µm and TiO_2_ nanopowders (anatase, particle size below 100 nm). The use of metal powders results in a larger specific surface area and, thus, in the case of silver, it allows greater release of silver ions and easier interaction with other particles, while providing better antimicrobial properties, which is an important factor when using these composites in antibacterial activities. Mixtures containing 5 wt% and 10 wt% TiO_2_ were prepared from the powders. Figure 1 below shows micrographs of the Ag and TiO_2_ powder particles and the Ag + 10 wt% TiO_2_ mixture. Silver powder has a dendritic and partially irregular shape, while TiO_2_ powder forms agglomerates composed of particles with a nearly spherical shape already at the stage of storage. In order to break the TiO_2_ clusters, mixing with the participation of balls was used, which resulted in a change in the shape and “spinning” of the silver powder particles. As a result of mixing in the Turbula T2F mixer with the participation of balls, a homogeneous mixture was obtained; the TiO_2_ particles are evenly distributed on the surface of the silver particles and do not form clusters. The parameters of the milling process were as follows: balls 5 mm in diameter, 100Cr6 heat-treated steel (about 50 HRC); milling time 270 min, input (milling media + mixture) was 40% of the milling cup volume; the ratio of the balls to the milled powder was 2:1.

**Figure 2:**
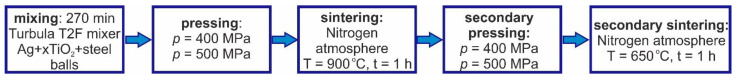
Scheme of Ag–xTiO_2_ composite manufacturing process.

The composites were produced in a single-action compaction press followed by sintering under nitrogen atmosphere at 900 °C for 1 h. Pressures of 400 MPa and 500 MPa were used for pressing, no lubricants were used, and they were cold pressed. In order to increase the density of the composites, the pressing operation was conducted at the same pressure and the samples were successively sintered under nitrogen atmosphere at the temperature of 650 °C for 1 h. The secondary pressing process was used to improve the density of the tested materials, the density of which after the first pressing was not sufficient. The second sintering is treated more as a procedure of eliminating internal stresses and recrystallization with a possible increase in the contact surface between the particles as a result of diffusion processes. The TiO_2_ reinforcement phase was added in amounts of 5 wt% and 10 wt%. Samples with dimensions of 4 × 5 × 40 mm were produced. The scheme of Ag–xTiO_2_ composite manufacturing process is presented in Figure 2.

Microstructure studies were carried out on the sinters made in this way using a Hitachi Su 70 scanning electron microscope (Hitachi, Tokyo, Japan). The micrographs were taken during operation of the detector collecting secondary electrons (SE) or backscattered electrons (BSE). Additionally, the chemical composition was analyzed using the wavelength-dispersive X-ray spectroscopy (WDS) method (Thermo Fisher Scientific, Waltham, MA, USA) and the phase composition was analyzed using a BRUKER X-ray diffractometer with a Co Kα = 0.179 nm (1.79 Å) cobalt lamp (D8 Advance, Bruker, Karlsruhe, Germany). Observations of the microstructure of the samples in the submicron range were made using a JEOL JEM-2010 ARP transmission electron microscope (Jeol Ltd., Tokyo, Japan). The specimens for observation were prepared by the ion thinning method using a Precision Ion Polishing System (PIPS) device by Gatan (Gatan, Inc., Pleasanton, CA, 94588, USA). Hardness measurements were made by the Brinell method employing an INNOVATEST hardness tester (Innovatest BV, Maastricht, The Netherlands). A tungsten carbide ball with a diameter of 2.5 mm was used and measurements were made with a load of 62.5 kg. Each sample was measured at five places, whereby a distance of at least two impression diameters was kept between the impressions. The flexural strength test was carried out using the three-point bending method on a Zwick Roell Z020 testing machine (Zwick AG, Ulm, Germany). The test was carried out in accordance with the PN EN ISO 7438 standard. The crosshead speed was 0.05 mm/s.

## 3. Results and Discussion

**Figure 3:**
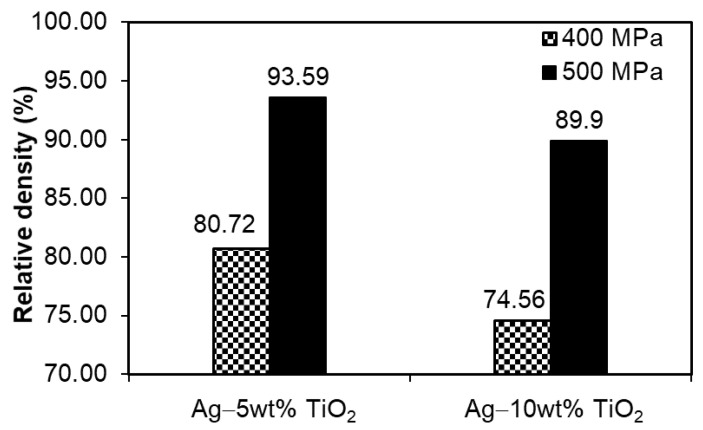
Relative densities of as-sintered PM silver matrix composites depending on chemical composition and process parameters (Archimedes method).

The final density of the composites largely depends on the first pressing pressure used, in addition to the proportion of TiO_2_ addition. A critical step in the pressing of silver-based materials in a multi-stage process is the first pressing; the second one only slightly changes or eliminates the swelling effect during the first sintering. Due to its hard nature, the addition of TiO_2_ makes the pressing process difficult, even if it has been successfully distributed on the surface of the silver particles by milling. Powder mixtures composed of two or more components exhibit poorer compatibility and formability than pure metals. The sintering of silver is a critical step in the entire manufacturing process of finished products owing to its tendency to swell during this process. In the studied materials, the TiO_2_ addition is evenly distributed in the sample, which makes it difficult to close the pores already at the pressing stage by creating a network of channels that allow the gas to move in the sintering atmosphere and, thus, prevent its pressure from increasing in the closed pores inside the sinter. As a result, it was possible to obtain a final sintered density at the level of about or higher than 90% for the samples that were pressed with the first pressure of 500 MPa. The graph presented in Figure 3 clearly shows that the difference between the pressing pressure of 400 and 500 MPa is significant and, despite the presence of a large number of through-pores, it does not favor their disappearance in the sinters compacted with lower pressure during sintering. An additional beneficial factor used during sintering is nitrogen atmosphere. Previously unpublished research by the team of authors revealed that silver sinters much better in nitrogen atmosphere and, with its high content in the material, it swells much less than in hydrogen atmosphere.

**Figure 4:**
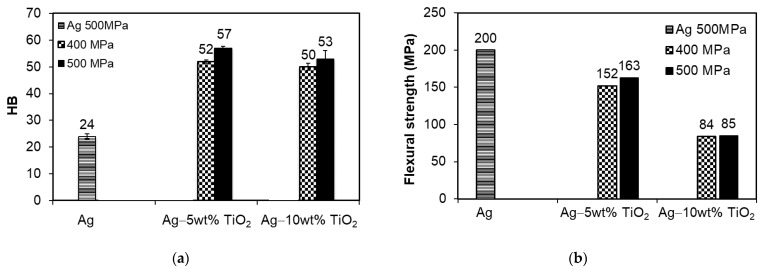
Results of testing composite properties: (**a**) Brinell hardness; (**b**) flexural strength.

As expected, the addition of TiO_2_ lowers the electrical conductivity of the tested materials, both in relation to solid copper and to silver pressed and produced under the same conditions (Table 1). The study shows that the content of the non-metallic phase is more important than the density obtained as a result of the production process. On the one hand, owing to the addition of TiO_2_, the electrical conductivity decreases, but, on the other hand, the hardness increases in relation to pure silver (Figure 4a), which significantly expands the possibilities of using such materials, e.g., in contacts operating in the start–stop cycle requiring higher hardness of contact surfaces. Increasing the TiO_2_ addition content does not increase the hardness. This is due to its distribution in the microstructure in the form of agglomerates and the higher porosity of these materials, which is the result of the increased content of the hard phase on the course of the pressing process, regardless of the pressure applied. Contrary to the hardness, the addition of the TiO_2_ reinforcing phase reduces the flexural strength (Figure 4b) and its effect is much greater than the porosity present in the sinters pressed at the pressure of 400 MPa. This is due to the way it is distributed on the boundaries of the silver particles, usually in the form of clusters, which facilitates the propagation of cracks through these areas.

**Figure 5:**
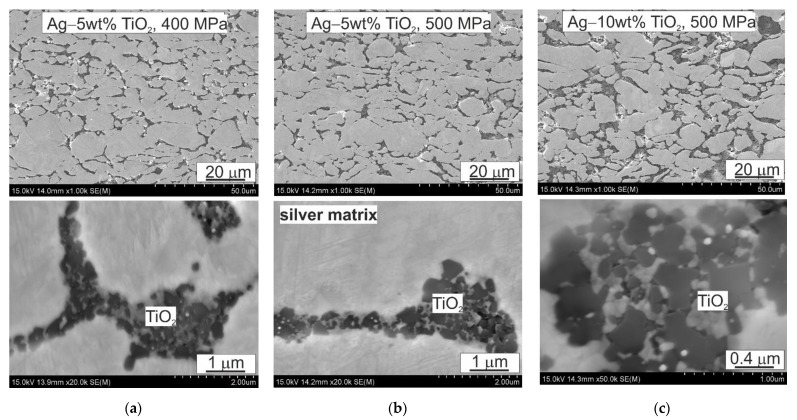
Composite microstructures: (**a**) Ag–5 wt% TiO_2_, *p* = 400 MPa; (**b**) Ag–5 wt% TiO_2_, *p* = 500 MPa; (**c**) Ag–10 wt% TiO_2_, *p* = 500 MPa; SEM.

The microstructure of the investigated materials (Figure 5) consists of areas of silver with single fine-grained TiO_2_ particles or clusters arranged on the boundaries. Fine pores are located locally at the interface between the silver particles themselves, as well as between the silver particles and clusters of TiO_2_. The observations of the microstructures confirmed that the applied milling is not sufficient to break down entirely all the clusters of TiO_2_ particles. The size of these clusters depends on the content of the reinforcing phase and their presence results from the tendency of fine powders to agglomerate, in addition to the difficulty of breaking these agglomerates at the stage of mixing the powders. The use of mixing balls only partially reduced the size of the agglomerations. Nevertheless, the observations with the scanning microscope and transmission electron microscopy confirm the good connection between the matrix and the reinforcing phase as well as the high coherence in the TiO_2_ clusters. A detailed analysis of the TiO_2_ regions reveals structural changes within its particles.

**Figure 6:**
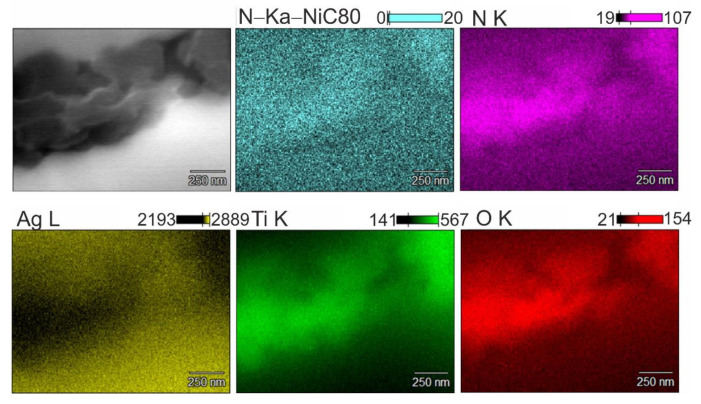
SEM and corresponding WDS mapping micrographs.

**Figure 7:**
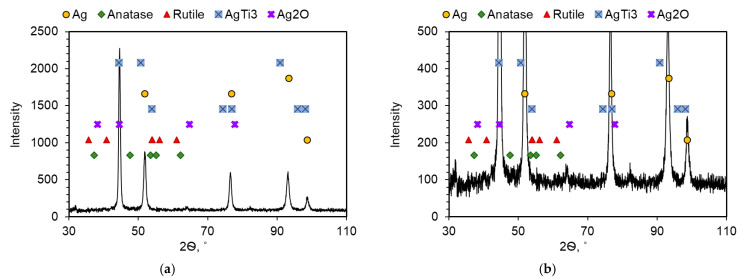
XRD analysis of Ag–5 wt% TiO_2_ composites; (**a**) in range of intensity 0–2500; (**b**) in range of intensity 0–500.

**Figure 8:**
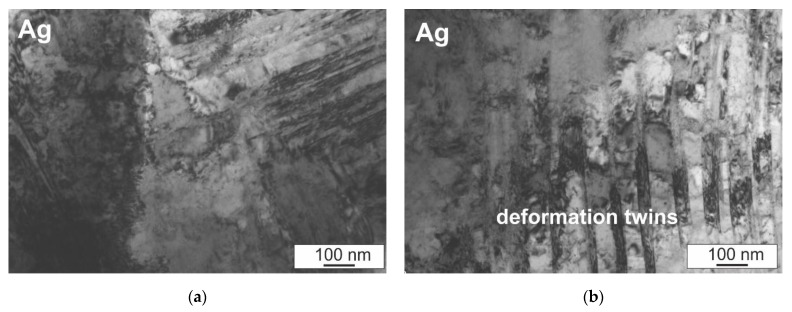

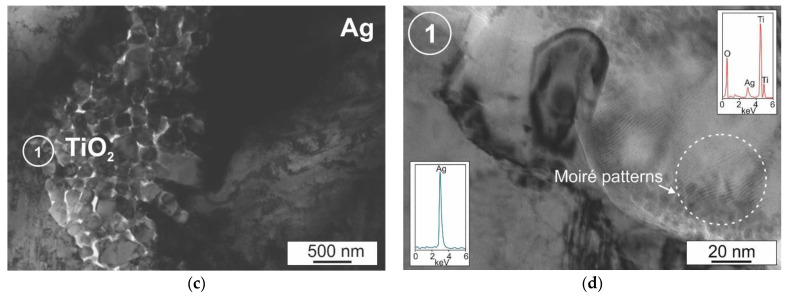
Microstructure of as-sintered Ag–5 wt% TiO_2_ composite, *p* = 500 MPa; (**a**,**b**) matrix; (**c**,**d**) Ag–TiO_2_ region; TEM.

The results of the chemical composition analysis by the WDS method indicate the diffusion of nitrogen into the area of the TiO_2_ reinforcing phase (Figure 6). The presence of Ag_3_N or AgN_3_ compounds was not found—the process was carried out under conditions that do not favor the formation of these compounds. The studies did not confirm the formation of Ti_x_N compounds either, despite the high affinity of titanium for nitrogen. The X-ray studies, the results of which are shown in Figure 7, showed the presence of a new AgTi_3_ phase which, after analyzing the element distribution maps, was located inside the TiO_2_ additive clusters, but close to the contact boundary with the silver matrix. This indicates the possibility of the release of metallic titanium, probably coming from the TiO_2_ particles, which, when analyzed by WDS, reveal oxygen-depleted areas inside. There is also the possibility of nitrogen diffusing inside the TiO_2_ particles from the sintering atmosphere in the titanium oxide reduction, leading to the formation of both the AgTi_3_ phase and nanometric silver oxide precipitates at the Ag–TiO_2_ interface, which results from the local excess oxygen in these areas and the sintering temperature, perhaps favoring its creation. This fact is confirmed by the observation and analysis of the chemical composition of these areas using TEM (Figure 8). The detailed analysis of the intensity peaks for TiO_2_ in Figure 7b suggests that some of it was converted to rutile at the sintering temperature and is consistent with the information on anatase, which is stable up to temperatures of around 800 °C.

**Figure 9:**
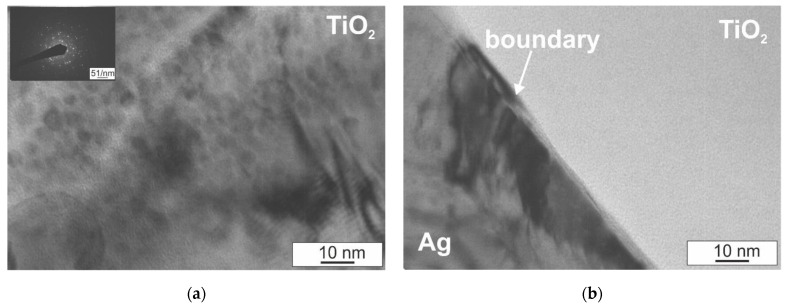
Microstructure of as-sintered Ag–TiO_2_ composite, *p* = 500 MPa; (**a**) TiO_2_; (**b**) boundary between silver matrix and TiO_2_ reinforcing phase; TEM.

In order to more fully verify the microstructure of the investigated composites, and, in particular, the bond of the silver matrix with the hard particles of the TiO_2_ reinforcing phase, observations of the microstructure were carried out using a transmission electron microscope. Selected results are presented in Figure 8 and Figure 9. In the silver matrix, against the background of cellular dislocation systems, deformation twins are present, which are visible in the form of bands of various widths not exceeding 100 nanometers and high dislocation density (Figure 8a,b). In metals with a face-centered cubic crystal structure, mechanical twinning is favored, among others, by the low stacking fault energy value of silver. In the case of the tested composites, the deformation twins probably arose during pressing and did not disappear despite sintering for 1 h at a temperature above the recrystallization temperature due to the presence of the hard TiO_2_ particles in the microstructure. The presence of the hard reinforcing phase particles prevents microstructure renewal processes, which favors a large accumulation of dislocations in the matrix area and the formation of characteristic twins in the form of bands. In addition, deformation twins are obstacles during dislocation slip, owing to which the matrix is characterized by a high dislocation density. The observations also confirmed the fact that a good bond of the silver matrix with the hard TiO_2_ phase was obtained and the local presence of nanometric silver oxide precipitates and AgTi_3_ precipitates were found, the growth of which was directed from the silver surface to the interior of the cluster located in this area (Figure 8c,d and Figure 9a,b). The presence of a strongly defective matrix around the presence of TiO_2_ particles and the increase in interfacial surfaces as a result of introducing the reinforcing phase will also act as a barrier to the movement of electrons, thus justifying the decrease in electrical conductivity for composites with a higher content of reinforcement in relation to silver.

Moiré patterns are also locally visible within the TiO_2_ particles (Figure 8d and Figure 9a), observed as parallel lines, formed by the interference of diffractive lattice planes, which overlap and may have different distances and/or orientations. Moiré patterns are produced by the superposition of two lattices with very small or equal spacings having a suitable mutual orientation [25].

The presence of a large number of defects, in addition to the reinforcing phase, is a component of the reinforcement of composites.

## 4. Conclusions

The conducted tests allowed the following conclusions to be formed:

- The applied conditions for the production of Ag–xTiO_2_ composites by means of powder metallurgy technology made it possible to obtain composites with a density exceeding 90%.
- The addition of TiO_2_ significantly reduces the electrical conductivity of the investigated materials, most strongly with the content of 10%, while improving the hardness of the composite, increasing the application possibilities of this type of material.
- Nitrogen, easily diffusible in the sintered material, can cause the appearance of free titanium (as a result of reduction), which reacts with silver to form AgTi_3_, and free oxygen atoms, together with silver from the matrix, form nanometric oxide, which locates on the border between silver and TiO_2_.

## Figures and Tables

**Table 1:** Electrical conductivity of Ag–xTiO_2_ composites.

Material	Electrical Conductivity, MS/m	Electrical Conductivity, % IACS
Ag	45.9	79.2
Ag-5 wt% TiO_2_ 400 MPa	32.6	56.2
Ag-5 wt% TiO_2_ 500 MPa	35	60.3
Ag-10 wt% TiO_2_ 400 MPa	18.4	31.8
Ag-10 wt% TiO_2_ 500 MPa	19.4	33.4

## Data Availability

Data sharing not applicable.
